# Yellow Pigments, Fomitellanols A and B, and Drimane Sesquiterpenoids, Cryptoporic Acids P and Q, from *Fomitella fraxinea* and Their Inhibitory Activity against COX and 5-LO

**DOI:** 10.3390/molecules18044181

**Published:** 2013-04-09

**Authors:** Kazuko Yoshikawa, Kazuaki Koso, Masumi Shimomura, Masami Tanaka, Hirofumi Yamamoto, Hiroshi Imagawa, Shigenobu Arihara, Toshihiro Hashimoto

**Affiliations:** Faculty of Pharmaceutical Sciences, Tokushima Bunri University, Yamashiro-cho, Tokushima 770-8514, Japan

**Keywords:** *Fomitella fraxinea*, Polyporaceae, fomitellanol, cryptoporic acid, cyclooxygenase-1 (COX-1) and COX-2, 5-lipoxygenase (5-LO)

## Abstract

Yellow pigments, fomitellanols A (**1a**) and B (**2a**), and drimane-type sesquiterpenoid ethers of isocitric acid, cryptoporic acids P (**3**) and Q (**4**), have been isolated fromthe fruiting bodies of *Fomitellafraxinea* (Polyporaceae). Their structures were established by a combination of extensive NMR spectroscopy and/or X-ray crystallographic analyses, and their biological activity against COX-1, COX-2, and 5-LO was investigated.

## 1. Introduction

**Figure 1:**
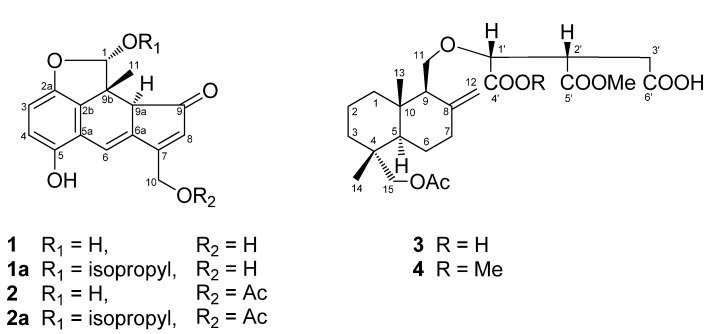
New compounds isolated in this work.

**Figure 2:**
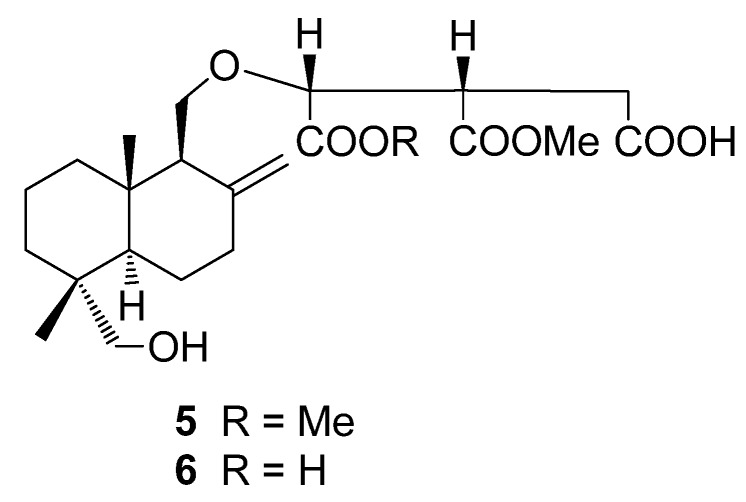
Known compounds isolated in this work.

In the course of our research aimed at the discovery of biologically active compounds from fungi, we previously studied the chemical constituents of four genera belonging to the Polyporaceae: *Laetiporus versisporus* [1], *Laetiporus sulphureus* var. *miniatus* [2], *Elfvingia applanata* [3], *Fomitopsis pinicola* [4], and *Daedalea dickisii* [5]. We subsequently initiated an investigation of *Fomitella fraxinea* (FR) Imaz. belonging to the same family. This fungus grows on dead trees in broad-leaved forests and is widely distributed in Japan [6,7]. Previous phytochemical studies on this fungus have led to the discovery of lanostane triterpenes, fomitellic acids A–D and their inhibitory activity against calf DNA polymerase α and rat DNA polymerase β [8], and a mannofucogalactan, fomitellan A, with a mitogenic effect [9]. The fruiting bodies of *F*. *fraxinea* were extracted with 70% isopropanol and the extract, after concentration, was dissolved in ethyl acetate. Fractionation of the EtOAc-soluble portion led to the isolation and characterization of four new compounds, which were designated as fomitellanols A (**1a**), B (**2a**) and cryptoporic acids P (**3**), and Q (**4**) (Figure 1), along with two known compounds, cryptoporic acids B (**5**) [10,11] and N (**6**) [12] (Figure 2). We describe here the isolation and structure elucidation of **1a**, **2a**, **3**, and **4** by extensive NMR and/or X-ray experiments, and the inhibitory activities of **1a**, **3**, **4**, and **6** against COX and 5-LO are also described. 

## 2. Results and Discussion

**Figure 3:**
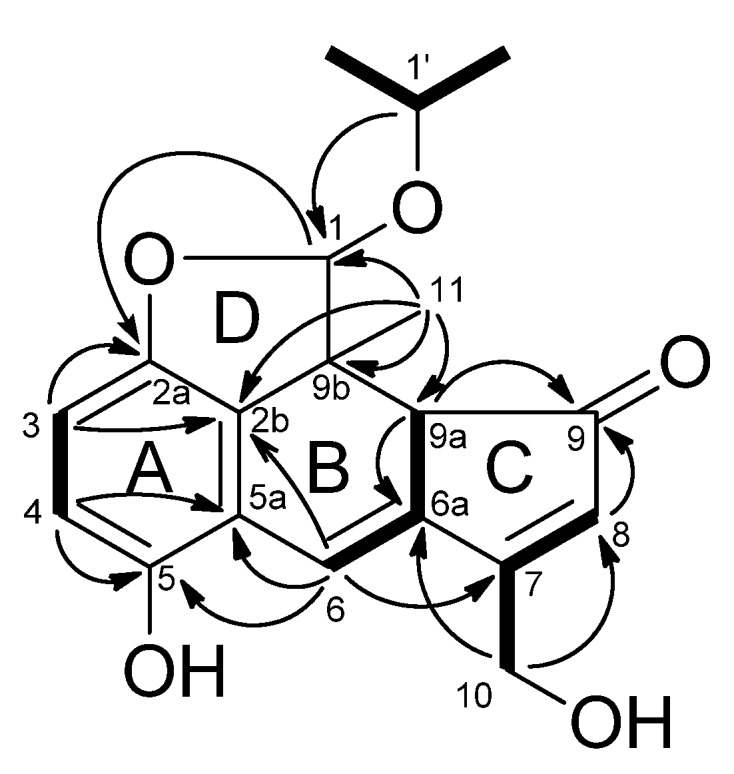
COSY (bold line) and selected HMBC (arrow line) of **1a**.

Fomitellanol A (**1a**), isolated as the 1-isopropoxy derivative of **1**, gave a molecular ion peak at *m/z* 327.1214 [M–H]^−^ (calcd. for C_19_H_19_O_5_, 327.1232) in its HRFABMS. This corresponds to a molecular formula of C_19_H_20_O_5_, requiring 10 degrees of unsaturation. The UV spectrum of **1a** showed absorption bands at 224, 291, 347, and 385 nm, indicating the presence of a conjugated system. The IR spectrum of **1a** showed absorptions at 3,250 cm^−1^ due to hydroxyl, and 1,670 cm^−1^ due to enone carbonyl groups. The ^1^H-NMR spectrum of **1a** exhibited one singlet methyl signal at δ 1.13 (s), one oxymethylene at δ 5.07 (dt, *J* = 17.3, 1.4 Hz) and 4.94 (dt, *J* = 17.3, 1.4 Hz), two methines at δ 5.95 (s), 4.10 (br. d, *J* = 1.9 Hz), two olefinic protons at δ 7.43 (d, *J* = 1.9 Hz), 6.91 (t, *J* = 1.4 Hz), and two AB-type aromatic protons at δ 6.97 (d, *J* = 8.5 Hz), 6.90 (d, *J* = 8.5 Hz). Further, one pair of equivalent secondary methyl signals at δ 1.31 (d, *J* = 6.3 Hz), 1.24 (d, *J* = 6.3 Hz), and one oxymethine at δ 4.26 (sept, *J* = 6.3 Hz) suggested the presence of an isopropoxy group. The 19 carbon signals observed in the ^13^C-NMR spectrum were sorted into three methyl, one oxymethylene (δ 58.9), three methines, two of which had an oxygen substituent (δ 113.6, 72.1); one sp^3^ quaternary carbon, ten sp^2^ carbons, four of which had proton substituents (δ 131.9, 115.9, 114.6, and 111.4); and a carbonyl carbon (δ 202.7) in combination with HMQC data. The planar structure of **1a** was constructed using the COSY and HMBC data. Namely, analysis of the COSY spectrum led to the four partial structures depicted by the bold lines, which were connected on the basis of the long-range correlations observed in the HMBC spectrum (Figure 3). 

The presence of ring C (3-hydroxymethyl-cyclopentaenone) was concluded from the HOH_2_C-C=CH spin system deduced from the COSY spectrum and HMBC correlations from H-8 (δ 6.91) and H-9a (δ 4.10) to C-9 (δ 202.7), from H-9a and H_2_-10 (δ 5.07, 4.94) to C-6a (δ 138.7), respectively. The construction of ring B and the junction of rings B and C were determined from the COSY correlations between H-6 (δ 7.43) and H-9a, and HMBC correlations from H_3_-11 (δ 1.13) to C-2b (δ 133.9), C-9a, C-9b (δ 46.2), from H-6 to C-2b, C-5a (δ 120.0), C-7 (δ 173.1). The construction of ring A and the junction of rings A and B were determined from the COSY correlation between H-3 (δ 6.90) and H-4 (δ 6.97), and HMBC correlations from H-3 to C-2a (δ 148.8), C-2b, from H-4 to C-5 (δ 150.2), C-5a, and from H-6 to C-5 (δ 150.2). Ring D fused to the hydroquinone ring A and cyclohexa-1,3-diene ring B was deduced from the following observations; HMBC correlations from H-1 (δ 5.95) to C-2a, from H_3_-11 to C-1 (δ 113.6), and further, from H-1' (δ 4.26) to C-1. 

**Figure 4:**
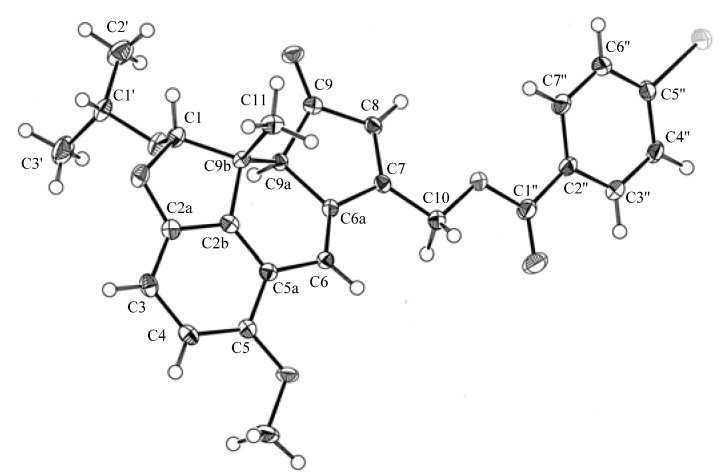
ORTEP drawing of the X-ray crystal structure of **1b**.

Thus, the planar structure of **1a** was determined to be 5-hydroxy-7-hydroxymethyl-1-isopropoxy-9b-methyl-9a,9b-dihydro-H-2-oxa-cyclopenta[d]acenaphthylen-9-one. The relative configurations of the three successive chiral centers at C-1, C-9a, and C-9b in **1a** were indicated by the following NOE analysis. The NOE between H_3_-11/H-1, H-1/H-1', and H-9a/H_3_-3' could establish 1S*, 9aS*, and 9bR* configurations. X-ray crystallographic analysis of **1b**, the corresponding *p*-bromobenzoate of **1a**, confirmed the proposed structure and established the three absolute configurations (Figure 4) [13].

Fomitellanol B (**2a**), isolated as the 1-isopropoxy derivative of **2**, gave an [M+Na]^+^ peak at *m/z* 393.1360 (calcd. for C_21_H_22_O_6_Na, 393.1338) in its HRFABMS, appropriate for a molecular formula of C_21_H_22_O_6_, which differed from the molecular formula of **1a** by the addition of 42 amu (C_2_H_2_O). The IR spectrum of **2a** showed absorptions for hydroxy (3,260 cm^−1^), carbonyl (1,740 cm^−1^), and enone carbonyl (1,670 cm^−1^) functions. The ^1^H-NMR and ^13^C-NMR data of **2a** were highly compatible with those of **1a**, the major difference being the presence of an acetyl group (δ_H_ 2.08; δ_C_ 20.4, 170.2) in **2a**. Comparison of the ^1^H-NMR data of **2a** and **1a** revealed the acylation shifts observed by +0.33 and +0.31 ppm at H_2_-10 (Table 1). Moreover, HMBC long-range correlations were observed between H_2_-10 (δ 5.38, 5.27) and the carbonyl carbon (δ 170.2) of the acetyl group. This set of acylation shifts and HMBC correlations indicated that **2a** was the C-10 acetyl analogue of **1a**. All other structural features of **2a** and **1a** were identical. Thus, the structure of **2a** was assigned as shown in Figure 1. 

**Table 1:** NMR data for fomitellanols A (1a) and B (2a) [600 MHz (1H) and 150 MHz (13C) in Pyridine-d5].

Position	1a		2a	
	δ_C_	δ_H_ (mult, *J* in Hz)	δ_C_	δ_H_ (mult, *J* in Hz)
1	113.6	5.95 (s)	113.4	5.93 (s)
2a	148.8		148.7	
2b	133.9		133.8	
3	111.4	6.90 (d, *J* = 8.5)	111.7	6.91 (d, *J* = 8.5)
4	115.9	6.97 (d, *J* = 8.5)	115.9	6.96 (d, *J* = 8.5)
5	150.2		150.2	
5a	120.0		119.8	
6	114.6	7.43 (d, *J* = 1.9)	115.5	7.38 (d, *J* = 1.9)
6a	138.7		137.9	
7	173.1		165.3	
8	131.9	6.91 (t, *J* =1.4)	133.2	6.54 (t, *J* =1.4)
9	202.7		202.1	
9a	49.4	4.10 (br. d, *J* = 1.9)	49.3	4.05 (br. s)
9b	46.2		46.2	
10	58.9	4.94 (dt, *J* = 17.3, 1.4)	60.4	5.27 (dt, *J* = 16.2, 1.4)
		5.07 (dt, *J* = 17.3, 1.4)		5.38 (dt, *J* = 16.2, 1.4)
11	16.5	1.13 (s)	16.5	1.10 (s)
1'	72.1	4.26 (sept, *J* = 6.3)	72.1	4.25 (sept, *J* = 6.3)
2'	22.2	1.24 (d, *J* = 6.3)	22.2	1.24 (d, *J* = 6.3)
3'	23.5	1.31 (d, *J* = 6.3)	23.5	1.31 (d, *J* = 6.3)
Ac			20.4	2.08 (s)
			170.2	

**Figure 5:**
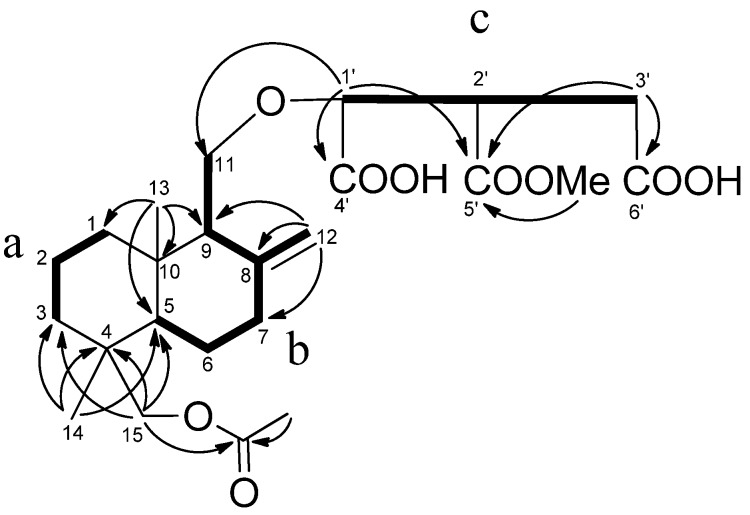
COSY (bold line) and selected HMBC (arrow line) of **3**.

Cryptoporic acidP (**3**) had a molecular formula of C_24_H_36_O_9_, determined by a quasimolecular ion peak at *m/z* 507.2003 (calcd. for C_24_H_36_O_9_K, 507.1996) in the HRFABMS, and requiring seven degrees of unsaturation. The IR spectrum showed adsorptions due to the carbonyl and hydroxyl groups at 3405, 1715, 1235, and 1035 cm^−1^. The ^1^H-NMR and ^13^C-NMR spectra of **3** exhibited two tertiary methyl groups (δ_Η_ 0.79; δ_C_ 15.9 and δ_Η_ 0.75; δ_C_ 17.5), one methoxy group (δ_Η_ 3.73; δ_C_ 51.9), two oxymethylenes (δ_Η _4.33, 3.81; δ_C_ 68.2 and δ_Η_ 3.94, 3.74; δ_C_ 73.0), one oxymethine (δ_Η_ 4.70; δ_C_ 80.1), *exo*-methylene (δ_Η_ 5.31, 5.02; δ_C_ 108.9 and δ_C_ 147.1), one ester carbonyl (δ_C_ 172.7), two carbonyls (δ_C_ 174.6, 173.7), in addition to one acetyl group (δ_Η_ 2.07; δ_C_ 170.1, 20.8) (Table 2). These data could account for five of seven degrees of unsaturation and the remaining two suggested two ring systems in **3**. The NMR data of **3** showed that its structure was closely related to the 15-hydroxyalbicanol ether of isocitric acid, cryptoporic acid N (**6**), obtained from the fungus *Cryptoporus sinensis* [12]. The complete assignment was unambiguously performed by 2D NMR spectra, including COSY, HMQC, HMBC, and ROESY data. The COSY correlations of **3** revealed the presence of three partial structures, a (H_2_-1/H_2_-2/H_2_-3), b (H-5/H_2_-6/H_2_-7/H_2_-12/H-9/H_2_-11), and c (H-1'/H-2'/H_2_-3'), which were connected by HMBC correlations from H_3_-14 (δ 0.75) to C-3, C-4, C-5, and C-15 (δ 73.0), from H_3_-13 (δ 0.79) to C-1, C-5, C-9, and C-10, from H_2_-12 (δ 5.31, 5.02) to C-7, C-8, and C-9, and from H_2_-15 (δ 3.94, 3.74) to C-3, C-4, C-5, and carbonyl carbon (δ_C_ 170.1) of the acetyl group, and revealed the presence of *exo*-methylene at C-8, oxymethylene at C-9, and acetoxy groups at C-15 in the drimane-type sesquiterpene skeleton shown in Figure 5. 

**Table 2:** NMR data for cryptoporic acidsP (**3**) and Q (4) [600 MHz (1H) and 150 MHz (13C) in Pyridine-d5].

Position	3		4	
	δ_C_	δ_H_ (mult, *J* in Hz)	δ_C_	δ_H_ (mult, *J* in Hz)
1	38.7	1.20 (m), 1.77 (m)	38.6	1.15 (m), 1.72 (m)
2	18.7	1.45 (m), 1.45 (m)	18.6	1.45 (m), 1.45 (m)
3	36.0	1.36 (m), 1.36 (m)	35.9	1.34 (m), 1.34 (m)
4	36.9		36.9	
5	49.3	1.40 (dd, *J* = 12.6, 2.2)	49.3	1.38 (m)
6	24.0	1.27 (m), 1.51 (m)	23.9	1.25 (m), 1.51 (m)
7	37.6	2.01 (m), 2.32 (m)	37.5	1.99 (m), 2.31 (m)
8	147.1		147.0	
9	56.2	2.21 (m)	56.0	2.15 (m)
10	38.9		38.8	
11	68.2	3.81 (dd, *J* = 9.6, 3.3)	68.6	3.70 (dd, *J* = 9.6, 3.3)
		4.33 (dd, *J* = 9.6, 8.2)		4.14 (dd, *J* = 9.6, 8.0)
12	108.9	5.02 (br. s)	108.6	4.98 (br. s)
		5.31 (br. s)		5.15 (br. s)
13	15.9	0.79 (s)	15.9	0.76 (s)
14	17.5	0.75 (s)	17.5	0.75 (s)
15	73.0	3.74 (d, *J* = 10.7)	73.0	3.73 (d, *J* = 11.0)
		3.94 (d, *J* = 10.7)		3.93 (d, *J* = 11.0)
1'	80.1	4.70 (d, *J* = 4.7)	79.5	4.57 (d, *J* = 4.4)
2'	45.7	4.14 (m)	45.6	3.98 (m)
3'	33.4	3.26 (dd, *J* = 17.0, 4.1)	33.6	3.09 (m)
		3.49 (dd, *J* = 17.0, 10.7)		3.35 (dd, *J* = 17.3, 9.3)
4'	173.7		171.5	
5'	172.7		172.1	
6'	174.7		175.0	
4'-OMe			51.9	3.65 (s)
5'-OMe	51.9	3.73 (s)	52.0	3.76 (s)
Ac	20.8	2.07 (s)	20.8	2.08 (s)
	170.1		170.1	

Further, the HMBC correlations from H-1' (δ 4.70) to C-11 (δ 68.2), C-4' (δ 173.7), C-5' (δ 172.7), from H_2_-3' (δ 3.26, 3.49) to C-5', C-6' (δ 174.7) indicated that an isocitric acid group was attached to C-11 through the ether linkage. The position of an ester group was determined at C-5' by HMBC correlations of H-1', H_2_-3', OMe (δ 3.73)/C-5' (Figure 5). In the ROESY spectrum, the methyl group at C-4 was confirmed β-orientation by NOEs between H_3_-13/H_3_-14, and H_3_-13/H_2_-11. The stereochemistry of **3** was established by the chemical transformation from **6**. Acetylation of **6** with Ac_2_O–pyridine afforded the monoacetate ([α]_D_^22^ +37.8). The spectral data of this monoacetate was identical to that of **3** ([α]_D_^22^ +33.6); therefore, the structure of **3**, including the absolute configuration, was identical to **6**, shown in Figure 1.

Cryptoporic acid Q (**4**) showed a quasimolecular ion peak [M–H]^−^ at *m/z* 481.2457 (calcd. for C_25_H_37_O_9_, 481.2437) in the HRFABMS, corresponding to the molecular formula C_25_H_38_O_9_. The ^1^H-NMR and ^13^C-NMR spectra of **4** were similar to those of **3**, except for the presence of one more methoxy group (δ_H_ 3.65, δ_C_ 51.9), and ester carbonyl carbon (δ 171.5), suggesting that **4** is the 15-acetoxyalbicanol ether of isocitric acid dimethyl ester. The positions of the two methoxy groups in the isocitric acid group were determined at C-4' and C-5' by the HMBC correlations observed from H-1' (δ 4.57)/C-4' (δ 171.5), OMe (δ 3.65)/C-4', and H-1'/C-5' (δ 172.1), H_2_-3' (δ 3.35, 3.09)/C-5', OMe (δ 3.76)/C-5'. The absolute configuration of **4** was finally resolved *via* acetylation of **5** to afford the corresponding acetate, whose NMR data and optical rotation ([α]_D_^22^ +25.6) were in good agreement with those of **4** ([α]_D_^22^ +20.9). Thus, cryptoporic acidQ was determined as shown in Figure 1. 

In this study, anti-inflammatory activities of isolated from this fungus were evaluated against a panel of key enzymes relating to inflammation, including COX-1, COX-2 and 5-LO in *in vitro* assays, as summarized in Table 3, Table 4. In the COX-1 and COX-2 assays, aspirin was used as a positive control. Compounds **1a** and **4** showed moderate activities (IC_50_ = 47.7 μM *vs.* 90.5% inhibition at 10 μg/mL, IC_50_ = 78.8 μM *vs.* 66.5% inhibition at 10 μg/mL) against COX-1, respectively; however, no isolated compounds had inhibitory activities against COX-2. In the 5-LO assay, compound **1** demonstrated an inhibitory effect on the formation of 5-HPETE with an IC_50_ value of 15.1 μM. A positive control used in this study, nordihydroguaiaretic acid (NDGA) showed an IC_50_ value of 0.4 μM.

**Table 3:** Inhibitory activity of **1a**, **3**, **4**, **6 **against COX enzymes.

Compound	% Inhibition of at 10 μg/mL		IC_50_^b,c^	
	COX-1	COX-2	COX-1	COX-2
Extract ^a^	96.4	59.0		
**1a**	90.5	7.5	47.7	ND ^d^
**3**	40.2	1.7	ND	ND
**4**	66.5	10.3	78.8	ND
**6**	35.8	19.0	ND	ND
**aspirin**	–	–	79.0	150

*^a^* EtOAc-soluble portion of 70% isopropanol of fruiting bodies of *F*. *fraxinea*. *^b^* IC_50_ values, in μM. *^c^* IC_50_ based on triplicate five-point titration. *^d^* ND: not determined.

**Table 4:** Inhibitory activity of **1a**, **3**, **4**, **6 **against 5-LO enzyme.

Compound	% Inhibition of at 10 μg/mL	IC_50_^b,c^
Extract ^a^	69.5	–
**1a**	84.7	15.1
**3**	14.9	ND ^d^
**4**	50.1	203.5
**6**	28.9	ND
**NDGA ^a^**	–	0.4

^a^ EtOAc-soluble portion of 70% isopropanol of fruiting bodies of *F. fraxinea*. ^b^ IC50 values, in μM. ^c^ IC50 based on triplicate five-point titration. *^d^* ND: not determined. *^e^* NDGA: nordihydroguaiaretic acid.

## 3. Experimental

### 3.1. General

Optical rotations were taken on a Jasco DIP-1030 polarimeter. UV spectra were recorded on a Shimadzu UV-1650PC, IR spectra were recorded on a Jasco FT/IR-5300, CD spectra were recorded on a Jasco J-725 and NMR spectra on a Varian Unity 600 spectrometer in C_5_D_5_N using TMS as an internal standard. NMR experiments included COSY, DEPT, HMQC, HMBC and ROESY. Coupling constants (*J* values) are given in Hz. The FABMS was measured on a JEOL JMS-700 mass spectrometer. Column chromatography was carried out on silica gel (230–400 mesh; Merck). Analytical TLC was performed on precoated Merck F_254_ silica gel plates and visualized by spraying with 30% H_2_SO_4_. HPLC was carried out on a Jasco PU-1580 pump equipped with a Jasco UV-970 detector and a Wakopack C30-5 column (5 μm, 20 mm i.d. × 250 mm, Wako). 

### 3.2. Materials

The fruit bodies were collected in Tokushima, Japan, in autumn 2008. A voucher specimen (TB 3085) is deposited in the Herbarium of Faculty of Pharmaceutical Sciences, Tokushima Bunri University, Tokushima, Japan. Material was identified by Dr. T. Hattori, the researcher of Forestry and Forest Products Research Institute.

### 3.3. Extraction and Isolation

The fresh fruit bodies (2.5 kg) of *F*. *fraxinea* were extracted with 70% IPA (8 L) at room temperature for 2 weeks. The IPA extract was partitioned between EtOAc and H_2_O. The EtOAc soluble portion (33.8 g) was subjected to silica gel column chromatography with *n*-hexane–EtOAc (9:1–0:10) to afford fractions 1–7. Fraction 2 (1.0 g) was passed through silica gel with *n*-hexane–EtOAc (7:3) and purified by preparative HPLC (ODS, 60% MeOH) to afford fomitellanol B (2a, 8.5 mg). Fraction 4 (5.6 g) was passed through silica gel with *n*-hexane–EtOAc (4:6–0:10) to afford fractions 4-1–5. Fractions 4-2 (0.2 g), and 4-4 (1.7 g) were successively purified by preparative HPLC (ODS, 55–75% MeOH) to afford fomitellanol A (1a, 48.6 mg), cryptoporic acids Q (4, 10.7 mg), and B (5, 15 mg) from fraction 4-2, and cryptoporic acid P (3, 18.2 mg) from fraction 4-4, respectively. Fraction 5 (4.6 g) was passed through silica gel with *n*-hexane–EtOAc (7:3) and purified by preparative HPLC (70–100% MeOH) to afford cryptoporic acid N (6, 20.8 mg).

*Fomitellanol* *A* (**1a**): Amorphous powder; [α]_D_^20^ +346 (*c* 0.42, MeOH); UV (MeOH) λ_max_ nm (logε): 224 (4.00), 291 (3.56), 347 (3.79), 385 (3.39); CD (MeOH) Δε nm: +7.63 (389), 328 (–3.05), 288 (+1.59), 268 (–0.86), 258 (+2.36), 239 (–8.32), 227 (+0.71), 222 (–0.86); FT-IR (dry film) ν_max_ cm^−1^: 3250 (OH), 1670 (C=O), 1075, 1030 (OH); ^1^H-NMR and ^13^C-NMR (C_5_D_5_N) see Table 1; HRFABMS *m/z* 327.1214 (Calcd. for C_19_H_19_O_5_, 327.1232).

*Fomitellanol B* (**2a**): Amorphous powder; [α]_D_^22^ +403 (*c* 0.14, MeOH); UV (MeOH) λ_max_ nm (logε): 221 (3.52), 292 (3.05), 348 (3.24); FT-IR (dry film) ν_max_ cm^−1^: 3260 (OH), 1740 (C=O), 1670 (C=O); ^1^H-NMR and ^13^C-NMR (C_5_D_5_N) see Table 1; HRFABMS *m/z* 393.1360 (Calcd. for C_21_H_22_O_6_Na, 393.1338).

*Cryptoporic acid* *P* (**3**): Amorphous powder; [α]_D_^22^ +33.6 (*c* 0.6, MeOH); FT-IR (dry film) λ_max_ cm^−1^: 3405 (OH), 1715 (C=O), 1235 (Ac), 1035 (OH); ^1^H-NMR and ^13^C-NMR (C_5_D_5_N) see Table 2; HRFABMS *m/z* 507.2003 (Calcd. for C_24_H_36_O_9_K, 507.1996).

*Cryptoporic acid* *Q* (**4**): Amorphous powder: [α]_D_^22^ +20.9 (*c* 0.40, MeOH): FT-IR (dry film) cm^−1^: 1730 (C=O), 1235 (Ac), 1035 (OH); ^1^H-NMR and ^13^C-NMR (C_5_D_5_N) see Table 2; HRFABMS *m/z* 481.2457 (Calcd. for C_25_H_37_O_9_, 481.2437).

3.4. p-Bromobenzoylation of **1a**

To a solution of **1a** (15 mg) in pyridine (2 mL) were added *p*-bromobenzoyl chloride (15 mg) and 4-dimethylaminopyridine (2 mg). The reaction mixture was stirred at room temperature for 24 h and then concentrated *in vacuo* to give a residue, which was purified by HPLC (ODS, 85% MeOH) to afford **1b** (5 mg) as yellow needles from diethyl ether–EtOH; mp 170–172 °C. X-ray crystallographic analysis confirmed the structure of **1b** (absolute configuration; ORTEP diagram, Figure 4).

### 3.5. X-ray Crystallographic Data for 1b

Single crystals of 1b, obtained by slow evaporation of MeOH, were selected, fitted onto a glass fiber, and measured at −173°C with a Bruker Apex II ultra diffractometer using Mo Kα radiation. Data correction and reduction were performed with the crystallographic package Apex II. The structures were solved by direct methods using SHELXS-97) and refined using full matrix least-squares based on *F*^2^with SHELXL-97. All non-hydrogen atoms were refined anisotropically, and hydrogen atoms were positioned geometrically. A total of 321 parameters were considered. Final disagreement indices were R1 = 0.0332 and wR2 = 0.0859 [I > 2 sigma (I)]. The ORTEP plot was obtained with the program PLATON. Crystal data: C_29_H_31_BrO_7_, MW = 571.45, monoclinic, space group *C2*, *Z* = 4, *a* = 28.078 (6) Å, *b* = 6.7924 (15) Å, *c* = 14.053 (3) Å, *β* = 100.059 (3)°. *V* = 2639.0 (10) Å^3^ [13]. 

### 3.6. Acetylations of 6 Giving 3 and of 5 Giving 4

Compounds **5** (10 mg) and **6** (10 mg) were acetylated overnight with Ac_2_O and pyridine (2 mL of each), respectively. The usual work-up afforded the acetates of **6** and **5**, which were identified by comparisons of their NMR data and optical rotations, as **3** and **4**, respectively.

### 3.7. COX-1 and COX-2-Catalyzed Prostaglandin Biosynthesis Assay in Vitro

Experiments were performed according to Futaki *et al.* [14] with modification. In brief, 200 U of COX-1 or COX-2 enzyme was suspended in 0.1 M Tris-HCl buffer (PH 7.5) containing hematin (1 μM) and phenol (2 mM), as co-factors. The reaction medium was preincubated with sample for 2 min at 37 °C, and 51.4 μM of [1,2,3,4,5,6,7,8,9,10,11,12,13,14] arachidonic acid (Sigma, St. Louis, MO, USA) was added and incubated for 2 min at 37 °C. To terminate the reaction and extract PGE_2_, 400 μL of *n*-hexane/EtOAc (2:1, V/V) was added to the reaction mixture and the preparation was centrifuged at 2,000 rpm for 1 min. The organic solvent phase was discarded. The extraction procedure was repeated twice, then 50 μL EtOH was added to the aqueous phase, and the preparation was at 2,000 rpm for 1 min. The amount of PGE_2_ was measured by radioimmunoassay using a liquid scintillation counter. COX-1 (EC1.14.99.1, isolated from ram seminal vesicles; Cayman Chemical Company, Ann Arbor, MI, USA) and COX-2 (isolated from sheep placenta, purity 70%; Cayman Chemical Company) were used. 

### 3.8. Measurement of RBL-1 5-Lipoxygenase Activity

The modified method of Blackman *et al.* [15] was used. Rat basophilic leukemia-1 (RBL-1) cells were grown in RPMI-1640 medium containing 10% heat-inactivated newborn calf serum (NCS), penicillin 100 units/mL, and streptomycin 100 mg/mL. Cells were cultured at 37 °C in 5% CO_2_. The assay system (0.5 mL) consisted of 50 mM phosphate buffer (pH 7.4), the test compound, 2 mM CaCl_2_, 0.66 mM arachidonic acid, and RBL-1 cell homogenate (1.5 × 10^7^ cells). Reaction mixtures were incubated at 37 °C for 3 min, and then MeOH (0.5 mL) was added to terminate the reaction. The mixture was centrifuged to remove the precipitated proteins, 5-HETE in the supernatant was analyzed by HPLC. The mixture was eluted through a Cosmosil 5C_18_ column (4.6 × 150 mm) at room temperature with 85% CH_3_CN at 235 nm. 

## 4. Conclusions

Two yellow pigments, fomitellanols A (**1a**) and B (**2a**) and drimane sesquiterpenoid ethers of isocitric acid, cryptoporic acids P (**3**) and Q (**4**), were isolated from the fruiting bodies of *Fomitella fraxinea* (Polyporaceae) together with two known compounds, cryptoporic acids B (**5**) and N (**6**). The structures of **1a**, **2a**, **3**, and **4** was determined by extensive spectroscopic analysis, and the absolute configuration was determined by X-ray analysis and/or the chemical transformations from **5** and **6**, respectively. Additionally, the known compounds, **5** and **6** are reported for the first time from *Fomitella fraxinea*. Compounds **1a** and **2a** were isolated as the 1-isopropoxy derivatives of **1** and **2**, respectively. They might possibly be artifacts derived from the reaction of the corresponding hemiacetals, with the isopropanol used in the extraction. Compounds **1a**, **3**, **4**, and **6** were tested for anti-inflammatory activities against COX-1, COX-2 and 5-LO in *in vitro* assays. It was assumed that compound **1** was most likely a dual inhibitor of the pathway involved in arachidonic acid metabolism in this study, although its activity is not apparent for COX-2 at the present. The result provides a potential explanation for the use of this fungus as herbal medicine in the treatment of inflammation, and it is potentially useful for developing new anti-inflammatory agents.
